# CONTRA-IL6: an interpretable hybrid convolutional neural network and Transformer framework for accurate prediction of interleukin-6-inducing peptides using protein language models

**DOI:** 10.1093/bib/bbag250

**Published:** 2026-06-11

**Authors:** Duong Thanh Tran, Nhat Truong Pham, Gwang Lee, Shaherin Basith, Balachandran Manavalan

**Affiliations:** Department of Integrative Biotechnology, College of Biotechnology and Bioengineering, Sungkyunkwan University, Suwon 16419, Gyeonggi-do, Republic of Korea; Department of Integrative Biotechnology, College of Biotechnology and Bioengineering, Sungkyunkwan University, Suwon 16419, Gyeonggi-do, Republic of Korea; Department of Physiology, Ajou University School of Medicine, Suwon 16499, Republic of Korea; Department of Molecular Science and Technology, Ajou University, Suwon 16499, Republic of Korea; Department of Physiology, Ajou University School of Medicine, Suwon 16499, Republic of Korea; Department of Integrative Biotechnology, College of Biotechnology and Bioengineering, Sungkyunkwan University, Suwon 16419, Gyeonggi-do, Republic of Korea

**Keywords:** IL-6-inducing peptides, deep learning, Transformer, convolutional neural networks, protein language model, immunotherapy

## Abstract

Interleukin-6 (IL-6) is a key immunomodulatory cytokine implicated in diverse physiological processes and pathological conditions, including autoimmune diseases, cancers, and cytokine storms. Immunogenic peptides capable of inducing IL-6 expression are key modulators of host immune responses and represent promising candidates for therapeutic design and epitope-based vaccine development. However, experimental identification of IL-6-inducing peptides remains laborious and unsuitable for large-scale screening. Although existing computational approaches show promise, many often struggle to capture both global contextual semantics and local motif-level features essential for peptide immunogenicity. To address these limitations, we present CONTRA-IL6, a novel deep learning framework that integrates Transformer fusion and convolutional localization modules with stacked pretrained protein language model embeddings to predict IL-6-inducing peptides. Comprehensive benchmarking on an independent dataset demonstrates that CONTRA-IL6 achieves superior predictive performance over six state-of-the-art predictors. Notably, it achieves the highest Matthews correlation coefficient (MCC, 0.504) and F1 (0.549) and improves over the best-performing existing method by 3.2% in MCC and 4.3% in F1, demonstrating balanced and robust performance. Feature space visualizations (uniform manifold approximation and projection, kernel density estimation) showed clear class separation, while 1D gradient-weighted class activation mapping++ highlighted strong attention to specific C-terminal regions. Crucially, we moved beyond these attribution methods by employing *in silico* mutagenesis, which causally confirmed the functional importance and physicochemical constraints. Ablation studies further confirmed the synergistic contribution of global and local modules to model performance. CONTRA-IL6 offers a robust, scalable, and interpretable solution for immunoinformatics research. The standalone package is freely available at https://pypi.org/project/contra-il6/ to facilitate broader community use.

## Introduction

The pleiotropic cytokine interleukin-6 (IL-6) plays a crucial role in regulating immune responses, inflammation, hematopoiesis, metabolism, and oncogenesis [1–3]. The structure of IL-6 comprises a four-helix bundle and is recognized by several alternative names, including interferon-β2, B-cell-stimulating factor-2, and hybridoma/plasmacytoma growth factor [4]. IL-6 is secreted by a diverse array of cell types, including monocytes, macrophages, dendritic cells, lymphocytes (T and B), fibroblasts, epithelial cells, keratinocytes, and endothelial cells, in response to pathogenic infection, tissue injury, or inflammatory stimuli [5, 6]. The biological functions of IL-6 occur through classical signaling, trans-signaling, and trans-presentation signaling pathways, which use the IL-6 receptor and glycoprotein 130 complex to mediate their effects. The signaling pathways lead to the activation of key transcription factors, including nuclear factor kappa B and signal transducer and activator of transcription 3 [7–9]. IL-6 plays a crucial role in the normal body by controlling acute-phase responses, differentiation, and B- and T-lymphocyte differentiation, immunoglobulin production, and Th2-type immune response promotion. Abnormal IL-6 expression leads to various acute and chronic diseases, including autoimmune disorders such as rheumatoid arthritis, systemic lupus erythematosus, multiple myeloma, immunoglobulin A nephropathy, cardiovascular diseases, and various cancers [10–12]. IL-6 functions as the primary cytokine in cytokine release syndrome, also known as “cytokine storm,” which mainly appears in severe infectious diseases, including COVID-19 [13]. Research shows that elevated IL-6 levels are directly linked to severe disease progression, multi-organ failure, and increased mortality rates in patients infected with severe acute respiratory syndrome coronavirus 2 (SARS-CoV-2) [14]. The SARS-CoV-2 spike protein activates mitogen-activated protein kinase signaling pathways, which leads to elevated IL-6 and soluble IL-6 receptor levels that produce intense inflammatory cascades, which worsen tissue damage and respiratory complications [15].

Recent evidence suggests that IL-6-inducing peptides (short amino acid sequences capable of eliciting IL-6 production) are crucial for understanding the dynamics of cytokine signaling in infectious and autoimmune diseases. These peptides often act as antigenic epitopes that interact with immune receptors to initiate IL-6-driven responses. Their identification is instrumental for dissecting the molecular mechanisms of IL-6 modulation and developing novel diagnostics, therapeutic targets, and epitope-based vaccines. However, traditional experimental approaches for identifying IL-6-inducing peptides are time consuming, resource intensive, and not scalable to large datasets. As a result, computational models using machine learning (ML) and deep learning (DL) have become indispensable for rapidly predicting IL-6-inducing peptides from large-scale sequence data, accelerating research in immunopathology and therapeutic design.

To date, several computational models have been developed to predict IL-6-inducing peptides. IL-6Pred [16] was among the first, employing 9149-dimensional (D) features reduced to 186 using support vector classification with L1 regularization, with a final Random Forest model trained on the top 10 features, achieving area under the receiver operating characteristic curves (AUCs) of 0.840 and 0.830 on training and independent test sets, respectively. StackIL6 [17] advanced this approach by integrating 12 feature descriptors and 5 ML algorithms through a stacking ensemble strategy, attaining an AUC of 0.841. MVIL6 [18] introduced a DL-based strategy by leveraging the MG-BERT [19] and Transformer [20] models to capture multi-view representations, thereby improving prediction accuracy. UsIL-6 [21] combined five peptide descriptors to form a 636-D feature vector, employed Boruta for feature selection, and used an Extremely Randomized Trees classifier, demonstrating superior performance over IL-6Pred and StackIL6 on independent datasets. RAG_MCNNIL6 [22] further advanced the field by integrating retrieval-augmented generation (RAG) with pretrained protein language model embeddings and a multi-window convolutional neural network (MCNN) architecture. This framework enriched peptide representations with relevant contextual information from homologous sequences, effectively learning multi-local motifs crucial for IL-6 induction. In parallel, DGIL-6 [23] introduced a structure-based DL model that leverages graph neural networks (GNNs) and predicted 3D peptide structures. By modeling peptides as graphs, where amino acids serve as nodes and structural adjacency determines edges, and incorporating features from the pretrained ESM-1b model, DGIL-6 applied a dual-channel GNN architecture combining Graph Attention Networks and Graph Convolutional Networks to capture detailed structural and sequential patterns. Although these methods achieved excellent performance during training, they exhibited several significant limitations. Most notably, these methods employed basic undersampling or oversampling to address class imbalance, which can discard useful data or lead to inflated performance during training. Furthermore, the final model of StackIL6 comprised 600 models for inference, making it computationally expensive. RAG_MCNNIL6, on the other hand, depends on an external similarity search in peptide databases, introducing not only inefficiency but also potential risks of dependency on external data quality. In addition, most existing methods lack interpretability, offering limited insights into the biological or sequence-level features that drive their predictions. Moreover, improving performance on independent datasets remains critical, especially by capturing both local immunogenic motifs and long-range peptide–epitope interactions essential for real-world applications.

To overcome these challenges, we propose CONTRA-IL6, a novel and interpretable framework for accurate prediction of IL-6-inducing peptides. CONTRA-IL6 employs a hybrid architecture that combines a Transformer Fusion (TF) module for capturing global contextual information and Convolutional Localization (CL) modules for identifying local motifs. CONTRA-IL6 integrates representations from multiple protein language models (PLMs), which are then optimized through top-K feature combination analysis and trained with Focal Loss (FL) [24] to effectively handle severe class imbalance. For interpretability, CONTRA-IL6 integrates 1D gradient-weighted class activation mapping++ (1D-Grad-CAM++) [25] to highlight key residues that drive model predictions. Through extensive benchmarking against six state-of-the-art methods (IL-6Pred, StackIL6, UsIL-6, MVIL6, DGIL-6, and RAG_MCNNIL6), CONTRA-IL6 demonstrated significant improvement across all key evaluation metrics. Uniform manifold approximation and projection (UMAP) [26] and kernel density estimation (KDE) further revealed distinct class separation, and attribution analyses aligned with prominent contributions from C-terminal regions. To move beyond descriptive attribution and rigorously establish the causal impact, we employed *in silico* mutagenesis (ISM) analysis to establish the causal relevance, confirming that these signals are driven by learned physicochemical constraints rather than dataset artifacts. Overall, these results show that CONTRA-IL6 is not only a highly accurate predictor but also a tool for generating testable, high-confidence hypotheses to guide therapeutic peptide design in IL-6-mediated diseases.

## Materials and methods

### Dataset construction

#### Benchmark dataset

We utilized the same benchmark dataset as in previous studies for fair comparison. This dataset, first introduced by Dhall *et al*. [16], was originally collected from Immune Epitope Database (IEDB) [27] and focused on human and mouse hosts. After removing all identical peptides and those longer than 25 amino acids, it comprises 365 experimentally validated IL-6 and 2991 non-IL-6-inducing peptides. Subsequently, this dataset was stratified and randomly split into 80%/20% ratio for training and independent evaluation. The training dataset consisted of 292 IL-6 and 2393 non-IL-6-inducing peptides, while the independent dataset consisted of 73 IL-6 and 598 non-IL-6-inducing peptides.

#### Case study dataset

We collected IL-6-inducing peptides from the IEDB using the search settings “Epitope: Linear peptide” and “Assay: T Cell.” Assays reporting IL-6 cytokine release were selected based on the outcome qualifier: peptides labeled “Positive” were defined as IL-6-inducing peptides, whereas peptides labeled as “Negative” were used as a non-inducing set. Thus, all negative samples were experimentally tested peptides that did not trigger IL-6 production. Duplicate sequences were removed, and only peptides with lengths between 8 and 25 amino acids were retained. To reduce redundancy and sequence similarity, clustering was performed using CD-HIT [28] with a sequence identity threshold of 0.7. After preprocessing and quality control, the final external dataset comprised 85 experimentally validated IL-6-inducing peptides and 135 experimentally validated non-inducing peptides. Detailed information regarding sequence sources is provided in Table S1, while the data collection pipeline and sequence diversity analyses are shown in Figs S1 and S2.

To further assess prospective validation beyond IEDB-derived data, we manually curated a specialized dataset of 19 food-derived peptides from literature published between 2024 and 2026. These peptides were obtained from diverse sources and experimentally tested for immunomodulatory activity, specifically IL-6 release, in the RAW264.7 and HaCaT cell lines. Unlike the training data, this dataset serves as a blind test of functional food candidates, assessing the model’s utility in real-world bioactive peptide discovery. Detailed sequences, sources, cell lines, and references are listed in Table S2.

Additionally, to support further evaluation of the model, more datasets were constructed from SARS-CoV-2 proteins using annotated data available from the National Center for Biotechnology Information SARS-CoV-2 resource (https://www.ncbi.nlm.nih.gov/sars-cov-2/). The dataset includes 1259 peptides from the spike protein, 61 peptides from the envelope protein, 208 from the membrane glycoprotein, 405 from the nucleocapsid phosphoprotein, 7082 from the open reading frame 1ab (ORF1ab) polyprotein, 261 from ORF3a, 47 from ORF6, 136 from ORF7a/7b, 107 from ORF8, and 24 peptides from ORF10.

### Feature extraction

In this study, we employed 12 different PLM-based features: Bepler [29], Word2Vec [30], SeqVec [31], FastText [32], GloVe [33], PLUS recurrent neural network (RNN) [34], ESM-1 [35], ESM-2 [36], and four ProtTrans [37] variants (ProtALBERT, ProtBERT, ProtXLNet, and ProtT5). While Word2Vec, FastText, and GloVe are traditional word-embedding techniques that generate static, fixed-dimensional embeddings based on word-co-occurrence statistics, the remaining models utilize advanced DL architectures, pretrained on extensive protein sequence databases.

We employed the *bio_embeddings* [38] package, which conveniently integrates all the aforementioned models into a unified framework. Table S3 summarizes the specific model variants used, including their versions, model types, model sizes, and embedding dimensions, while detailed descriptions can be found in the Supplementary materials.

### Framework of CONTRA-IL6

**Figure 1:**
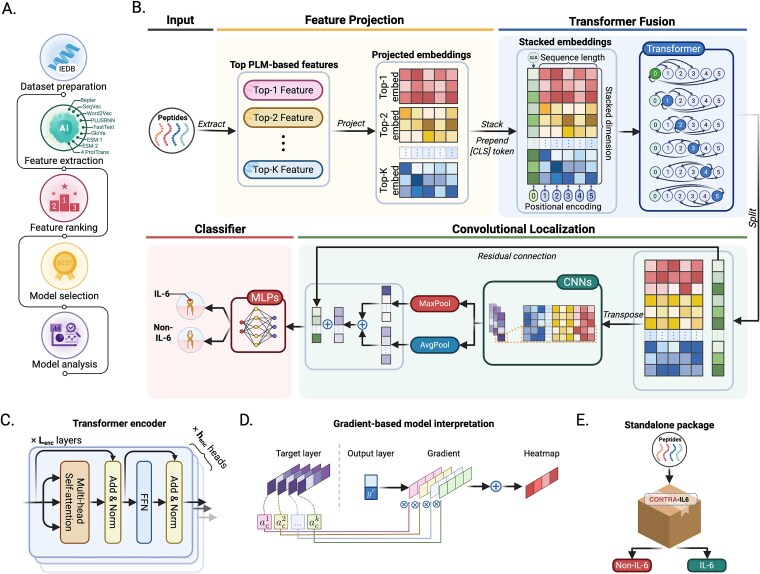
An overview of the CONTRA-IL6 framework. (A) End-to-end pipeline including dataset preparation from the Immune Epitope Database, extraction and ranking of 12 protein language model–based features, selection of the best model through stacking top-*K* features, and model analysis. (B) The CONTRA-IL6 architecture comprises Feature Projection, Transformer Fusion, Convolutional Localization, and Classifier modules. (C) Detailed view of the Transformer encoder. (D) Gradient-based model interpretation using one-dimensional gradient-weighted class activation mapping++. (E) Standalone package for accessibility.

Figure 1 illustrates the overview of our CONTRA-IL6 framework, which consists of four main modules: (i) Feature Projection (FP), (ii) TF, (iii) CL, and (iv) Classifier module. The details of these modules are presented below:

#### FP module

Let us consider a list of $K$ ranked PLM-based features, denoted as ${\left\{{A}_i\right\}}_{i=1}^K\in{\mathbb{R}}^{L\times{d}_{A_i}}$, where $L$ represents the sequence length and ${d}_{A_i}$ denotes the embedding dimension of feature ${A}_i$.

To effectively integrate these top-$K$ features, we explore two stacking strategies: uniform projection and proportional projection. In the uniform projection strategy, each feature is projected to a shared target dimension ${d}_{\mathrm{target}}$, after which they are concatenated. In contrast, the proportional projection strategy allocates dimensions to each feature such that the total dimensionality of the stacked representation equals ${d}_{\mathrm{target}}$.

The first approach, while straightforward, does not optimize computational efficiency, which means its cost increases substantially as $K$ grows. The second approach offers a more scalable solution by utilizing the feature space more effectively and minimizing both redundancy and computational overhead. Figure S3 illustrates the growth in model complexity, measured by the number of parameters and floating-point operations for both of the aforementioned approaches, providing a clearer comparison. Consequently, we adopted the second approach as our final projection strategy. The projected features are given by


(1) \begin{align*} &{\left\{{A}_i^{\mathrm{prj}}\right\}}_{i=1}^K = \mathrm{Linear}\left({A}_i\right),{A}_i^{\mathrm{prj}}\in{\mathbb{R}}^{L\times{d}_{A_i^{\mathrm{prj}}}},\mathrm{where}\ {d}_{A_i^{\mathrm{prj}}} \nonumber \\&= \left\{\begin{array}{@{}l@{}}\left\lfloor \frac{d_{\mathrm{target}}}{K}\right\rfloor, \mathrm{for}\ i\in \left\{1,2,\cdots, K-1\right\}\\{}\left\lfloor \frac{d_{\mathrm{target}}}{K}\right\rfloor +\left({d}_{\mathrm{target}} \operatorname{mod}\ K\right),\mathrm{for}\ i=K\ \end{array}\right. \end{align*}

Here, if ${d}_{\mathrm{target}}$ is not evenly divisible by $K$, the remaining dimensions are assigned to the last feature, ensuring that the total projection remains consistent with ${d}_{\mathrm{target}}$ while maintaining feature representation integrity.

#### TF module

After obtaining a set of projected features ${A}_i^{\mathrm{prj}}$, in this module, we stacked them together and prepended a learnable special embedding, denoted as $\mathrm{CLS}\in{\mathbb{R}}^{1\times{d}_{\mathrm{target}}}$, to form the final input sequence ${A}_{\mathrm{stacked}}$. This is defined as


(2) \begin{equation*} {\displaystyle \begin{array}{c}{A}_{\mathrm{stacked}}=\mathrm{concat}\left(\mathrm{CLS},\mathrm{stack}\left({A}_i^{\mathrm{prj}}\right)\right),{A}_{\mathrm{stacked}}\in{\mathbb{R}}^{\left(L+1\right)\times{d}_{\mathrm{target}}}\ \end{array}} \end{equation*}

The inclusion of the classification ($\mathrm{CLS}$) token is inspired by the previous methods [39–41], where a special token is prepended to input sequences during pretraining to capture a holistic representation of the entire sequence, particularly useful for classification tasks. In our approach, the CLS embedding serves a similar purpose, enabling the model to learn a global contextual representation of the peptide sequence and facilitating more effective information aggregation in downstream processing.

Following the stacking step, ${A}_{\mathrm{stacked}}$ is added with learnable positional embeddings $\mathrm{P}{\mathrm{E}}_{\mathrm{stacked}}\in{\mathbb{R}}^{\left(L+1\right)\times{d}_{\mathrm{target}}}$, yielding the input to the Transformer encoder [20]:


(3) \begin{equation*} {\displaystyle \begin{array}{c}{H}^{(0)}={A}_{\mathrm{stacked}}+\mathrm{P}{\mathrm{E}}_{\mathrm{stacked}}\end{array}} \end{equation*}

This input is then passed through ${L}_{\mathrm{enc}}$ layers of Transformer encoders, defined recursively as


(4) \begin{equation*} {\displaystyle \begin{array}{c}{H}^{(l)}={\mathrm{TransformerLayer}}^{(l)}\left({H}^{\left(l-1\right)}\right),\kern0.5em \forall l\in \left\{1,2,\cdots, {L}_{\mathrm{enc}}\right\}\end{array}} \end{equation*}

Let ${\overset{\sim }{A}}_{\mathrm{stacked}}={H}^{\left({L}_{\mathrm{enc}}\right)}$ denote the final output of the Transformer encoder. Each Transformer layer consists of a multi-head self-attention (MHSA) [20] mechanism, layer normalization (LN), and a feed-forward network (FFN), formulated as


(5) \begin{equation*} {\overset{\sim }{H}}^{(l)}= LN\left({H}^{\left(l-1\right)}+ MHSA\left({H}^{\left(l-1\right)}\right)\right),\end{equation*}

(6) \begin{equation*} {\displaystyle \begin{array}{c}{H}^{(l)}= LN\left({\overset{\sim }{H}}^{(l)}+ FFN\left({\overset{\sim }{H}}^{(l)}\right)\right).\end{array}} \end{equation*}

After encoding, we extract the CLS embedding from ${\overset{\sim }{A}}_{\mathrm{stacked}}$, denoted as ${F}_{\mathrm{global}}\in{\mathbb{R}}^{d_{\mathrm{target}}}$, which is later utilized as a residual connection. The remaining token embeddings, denoted as ${\overset{\sim }{A}}_{\mathrm{seq}}\in{\mathbb{R}}^{L\times{d}_{\mathrm{target}}}$, are refined in the subsequent module.

#### CL module

While the TF module, described in the previous section, captures the global contextual representation of the peptide sequence, this module is designed to extract local contextual patterns. To achieve this, we leverage 1D convolutional (Conv1D) [42] layers, which are well suited for detecting local motifs by applying filters over adjacent elements in the sequence. Unlike fully connected layers that consider the entire input simultaneously, Conv1D layers operate over localized receptive fields, enabling efficient extraction of motif-like patterns that may be crucial for downstream tasks.

In our approach, we employ a two-layer convolutional architecture, formulated as 


(7) \begin{equation*} {B}_{\mathrm{seq}}=\sigma \left({W}_1^{\mathrm{conv}}\blacklozenge{\overset{\sim }{A}}_{\mathrm{seq}}^{\top }+{b}_1\right),\kern0.5em {B}_{\mathrm{seq}}\in{\mathbb{R}}^{\frac{d_{\mathrm{target}}}{2}\times{L}_{\mathrm{out}}^{(1)}}, \end{equation*}

(8) \begin{equation*} {B}_{\mathrm{seq}}^{\prime }=\sigma \left({W}_2^{\mathrm{conv}}\blacklozenge{B}_{\mathrm{seq}}+{b}_2\right),\kern0.5em {B}_{\mathrm{seq}}^{\prime}\in{\mathbb{R}}^{d_{\mathrm{target}}\times{L}_{\mathrm{out}}^{(2)}}. \end{equation*}

Here, ${\overset{\sim }{A}}_{\mathrm{seq}}^{\top }$ denotes the transposed form of the encoded sequence features to match the expected input shape for Conv1D. The convolutional filters are defined as ${W}_1^{\mathrm{conv}}\in{\mathbb{R}}^{\frac{d_{\mathrm{target}}}{2}\times{d}_{\mathrm{target}}\times k}$ and ${W}_2^{\mathrm{conv}}\in{\mathbb{R}}^{d_{\mathrm{target}}\times \frac{d_{\mathrm{target}}}{2}\times k}$, where $k$ is the kernel size. The operator $\blacklozenge$ denotes the 1D convolution operation, applied with stride $s$, dilation $d$, and padding $p=\frac{k}{2}$. The terms ${b}_1$ and ${b}_2$ represent learnable biases, and $\sigma$ is the rectified linear unit activation function.

The output sequence lengths after each convolutional layer, ${L}_{\mathrm{out}}^{(1)}$ and ${L}_{\mathrm{out}}^{(2)}$, are computed as 


(9) \begin{align*} {L}_{\mathrm{out}}^{(1)}&=\left\lfloor \frac{L+2p-d\left(k-1\right)-1}{s}+1\right\rfloor, \nonumber \\{L}_{\mathrm{out}}^{(2)}&=\left\lfloor \frac{L_{\mathrm{out}}^{(1)}+2p-d\left(k-1\right)-1}{s}+1\right\rfloor. \end{align*}

Following the convolutional operations, we apply max pooling and average pooling along the sequence dimension to extract compact feature representations. These operations aggregate sequence information by capturing both salient high-activation signals and overall contextual distributions:


(10) \begin{equation*} {B}_{\mathrm{max}}=\mathop{\max }\limits_{i\in \left\{1,\cdots, {L}_{\mathrm{out}}^{(2)}\right\}} {B}_{\mathrm{seq}}^{\prime }(i),\kern0.5em {B}_{\mathrm{max}}\in{\mathbb{R}}^{d_{\mathrm{target}}},\end{equation*}

(11) \begin{equation*} {B}_{\mathrm{avg}}=\frac{1}{L_{\mathrm{out}}^{(2)}}\sum_{i=1}^{L_{\mathrm{out}}^{(2)}}{B}_{\mathrm{seq}}^{\prime }(i),\kern0.5em {B}_{\mathrm{avg}}\in{\mathbb{R}}^{d_{\mathrm{target}}}.\end{equation*}

We then combine the two pooled vectors to form the dual pooling fusion as the final local feature representation:


(12) \begin{equation*} {\displaystyle \begin{array}{c}{F}_{\mathrm{local}}=\frac{B_{\mathrm{max}}+{B}_{\mathrm{avg}}}{2},\kern0.5em {F}_{\mathrm{local}}\in{\mathbb{R}}^{d_{\mathrm{target}}}\end{array}} \end{equation*}

This fusion strategy balances high-activation motif signals and smooth contextual patterns, producing a robust local representation for downstream processing.

#### Classifier module

In the final module of the framework, we integrate the global and local representations via a residual connection to form a unified feature representation. Specifically, the global contextual embedding ${F}_{\mathrm{global}}$ and the local feature representation ${F}_{\mathrm{local}}$ are fused to capture both sequence-wide dependencies and localized motif patterns. This combined representation serves as the input to an FFN, which produces the final logits for binary classification between IL-6-inducing and no-IL-6-inducing peptides.

The process is formulated as follows:


(13) \begin{equation*} {F}_{\mathrm{final}}={F}_{\mathrm{global}}+{F}_{\mathrm{local}},\kern0.5em {F}_{\mathrm{final}}\in{\mathbb{R}}^{d_{\mathrm{target}}},\end{equation*}

(14) \begin{equation*} {C}_{\mathrm{class}}=\mathrm{FFN}\left({F}_{\mathrm{final}}\right),\kern0.5em {C}_{\mathrm{class}}\in{\mathbb{R}}^2. \end{equation*}

This design ensures that both global semantics and fine-grained patterns are jointly considered in the final classification, enhancing the model’s predictive performance. Furthermore, details of the optimization process, including loss function and hyperparameter optimization, are provided in the Supplementary materials, Table S4 and Fig. S4.

## Results and discussion

### Construction of baseline models for feature ranking

**Figure 2:**
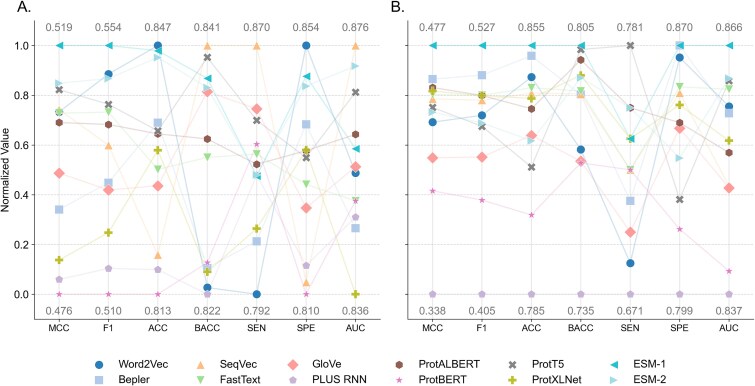
Performance comparison between 12 single protein language model-based features. (A) Performance evaluation on cross-validation datasets. (B) Performance evaluation on the independent dataset.

To evaluate and rank the predictive power of different PLM-based features, we constructed a series of baseline models using 12 individual feature representations as mentioned in the previous section. Each model was trained using our proposed architecture with $K=1$, i.e. only a single feature type was used at a time. For each feature representation, we selected the best-performing model by tuning hyperparameters and assessing performance through stratified 10-fold cross-validation. Figure 2 presents the performance comparison of these single-feature models on both the cross-validation set and the independent test set, using their respective optimal hyperparameters. In the figure, the value of each metric is scaled from 0 (the worst feature’s value) to 1 (the best feature’s value).

On the cross-validation set (Fig. 2A), ESM-1 achieved the highest overall performance, with a Matthews correlation coefficient (MCC) of 0.519 and an F1 score (F1) of 0.554. These values represent a 0.7%–4.3% improvement in MCC and a 0.5%–4.4% improvement in F1 compared to the other embedding models. ESM-2 and ProtT5 closely followed, with MCCs of 0.512 and 0.511 and F1s of 0.548 and 0.544, respectively. In terms of local metrics, SeqVec achieved the highest sensitivity (SEN) (0.870) and the AUC (0.876), indicating its ability to detect IL-6-inducing peptides. However, it exhibited relatively lower specificity (SPE) (0.812) and MCC (0.508), suggesting some limitations in accurately identifying non-IL-6 peptides. In contrast, Word2Vec achieved the highest SPE (0.854), but at the expense of SEN (0.792), limiting its effectiveness in identifying IL-6-inducing peptides. At the lower end of performance, ProtBERT, PLUS RNN, and ProtXLNet achieved MCCs of 0.476, 0.479, and 0.482, respectively, suggesting their moderate discriminative ability.

Evaluation on the independent test set (Fig. 2B) revealed a slightly different performance trend. Nevertheless, ESM-1 consistently outperformed all other models, achieving the highest scores across multiple metrics: MCC (0.477), F1 (0.527), accuracy (ACC) (0.855), balanced ACC (BACC) (0.805), SPE (0.870), and AUC (0.866). These results reflect substantial improvements of 1.9%–13.9% in MCC, 1.5%–12.2% in F1, 0.2%–7.0% in ACC and BACC, 0.4%–7.1% in SPE, and 0.4%–2.9% in AUC over the other models, highlighting ESM-1’s robustness and strong generalizability to unseen data. Bepler ranked second on the independent test set with an MCC of 0.458 and an F1 of 0.512, but the trend did not align with its cross-validation performance. Conversely, ProtBERT and PLUS RNN consistently underperformed with MCCs of 0.396 and 0.338, respectively, indicating limited generalizability. While ESM-2 and ProtT5 ranked second and third in cross-validation, their performance on the independent set was MCCs of 0.452 and 0.440 and F1s of 0.502 and 0.489, respectively. This discrepancy suggests that although these models generalize reasonably well, their performance may be partially over-optimized to the training distribution, potentially capturing specific patterns that do not hold as strongly in unseen data.

Based on MCC performance from cross-validation, the final ranking of the 12 models is as follows: ESM-1, ESM-2, ProtT5, SeqVec, FastText, Word2Vec, GloVe, Bepler, ProtXLNet, PLUS RNN, and ProtBERT. This ranking serves as the foundation for the subsequent feature stacking and selection process for constructing the optimal model configuration within the CONTRA-IL6 framework.

### Development of CONTRA-IL6

**Figure 3:**
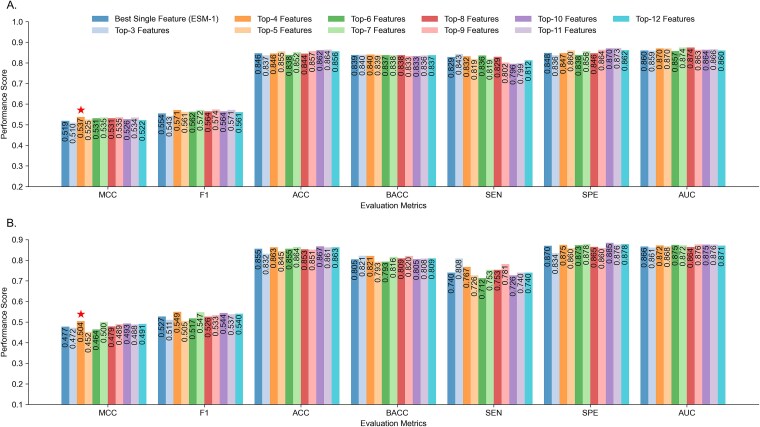
Performance comparison across top-*K* experiments with *K* ranging from 3 to 12. (A) Performance evaluation on cross-validation datasets. (B) Performance evaluation on an independent dataset. The star symbol (★) indicates the best performer, based on the Matthews correlation coefficient.

To construct the final CONTRA-IL6 model, we systematically investigated the optimal integration of diverse protein feature representations. In this approach, we progressively incorporated the top-$K$ performing features, where $K$ ranged from 3 to 12, ranked by their individual cross-validation performance as described in the previous section. The resulting performance was compared against the best-performing single feature, ESM-1 (Fig. 3). On the cross-validation dataset, integrating the top-3 features (ESM-1, ESM-2, and ProtT5) led to a slight decline in performance compared to using only ESM-1 (Fig. 3A), with the MCC decreasing from 0.519 to 0.510, the F1 from 0.554 to 0.543, and the AUC from 0.860 to 0.859. However, the addition of SeqVec at $K=4$ improved the prediction performance. The MCC notably increased, achieving a range of 0.522–0.537 (representing a 0.3%–1.8% improvement), the F1 performance with a range of 0.561–0.571 (0.7%–1.7% improvement), and the AUC reached in the range of 0.863–0.874 (0.3%–1.4% improvement). The top-4 configuration achieved the peak overall performance with an MCC of 0.537, F1 of 0.571, BACC of 0.840, and AUC of 0.870. Beyond the optimal top-4 threshold, further integration of features (from $K=5$ to $K=12$), no substantial gains were observed, indicating that performance gains plateaued beyond the optimal feature combination.

Performance trends on the independent test dataset remarkably mirrored those observed during the cross-validation, underscoring the generalizability of our models (Fig. 3B). Furthermore, training curves across the 10-fold cross-validation demonstrated stable convergence and consistent validation performance, highlighting the robustness of the training process (Fig. S5). Consistent with the cross-validation results, the top-3 feature combination resulted in a marginal performance decrease compared to the single best feature. Conversely, models incorporating four or more features consistently outperformed the baseline model, with the MCC increasing by 0.2%–2.7%, F1 by 0.6%–2.2%, and AUC by 0.2%–1.0%. Notably, the top-4 feature combination again demonstrated the highest performance on the independent dataset, confirming its robustness and generalizability across unseen data. As observed in cross-validation, performance plateaued from $K=5$ to $K=12$, showing no further meaningful improvements.

In conclusion, these results demonstrate that the top-4 feature combination provides the optimal balance between feature diversity, informational content, and predictive efficiency. The synergistic strength of complementary protein embeddings enables CONTRA-IL6 to outperform both single feature and other hybrid features. Based on its consistently superior and stable performance across both cross-validation and independent datasets, the top-4 feature combination was selected as the final architecture for CONTRA-IL6, ensuring the robust discrimination of IL-6-inducing and non-IL-6-inducing peptides.

### Performance comparison with existing methods

To rigorously establish the predictive superiority of CONTRA-IL6, we conducted a comprehensive comparative analysis against six state-of-the-art IL-6-inducing peptide prediction tools, including IL-6Pred [16], StackIL6 [17], UsIL-6 [21], MVIL6 [18], DGIL-6 [23], and RAG_MCNNIL6 [22]. Among these, only IL-6Pred and RAG_MCNNIL6 provide publicly accessible tools. Consequently, we evaluated these tools directly on the independent dataset to ensure transparency in reproducing the results of these methods. For non-reproducible methods, performance values were taken from the original publications because executable tools or source codes were not publicly available. Notably, all methods, including CONTRA-IL6, were trained and evaluated on the same benchmark datasets originally curated by Dhall *et al*. [16], ensuring that cross-study comparisons remain grounded in a common experimental framework. Table 1 shows that CONTRA-IL6 achieved the highest MCC of 0.504, demonstrating significant improvements ranging from 3.2% to 18% over existing methods. This notable gain underscores CONTRA-IL6’s superior capability to discriminate between IL-6-inducing and non-inducing peptides. In particular, CONTRA-IL6 consistently surpassed RAG_MCNNIL6 (0.442), DGIL-6 (0.472), MVIL6 (0.469), UsIL-6 (0.445), StackIL6 (0.393), and IL-6Pred (0.324), demonstrating a stronger overall correlation between predicted and actual labels.

**Table 1:** Performance comparison between CONTRA-IL6 and existing methods on the independent dataset.

Model	Reproducible?	MCC	F1	ACC	BACC	SEN	SPE	AUC
IL-6Pred [16]	Yes	0.324	0.382	0.735	0.743	0.753	0.732	0.830
StackIL6 [17]	No	0.393	0.428	0.753	0.795	0.849	0.741	0.841
UsIL-6 [21]	No	0.445	0.487	0.818	0.808	0.795	0.821	0.870
MVIL6 [18]	No	0.469	0.498	0.811	0.834	0.863	0.804	0.883
DGIL-6 [23]	No	0.472	0.498	0.808	**0.838**	**0.877**	0.799	**0.902**
RAG_MCNNIL6 [22]	Yes	0.442	0.506	**0.875**	0.749	0.589	**0.910**	0.804
CONTRA-IL6 (Ours)	Yes	**0.504**	**0.549**	0.863	0.821	0.767	0.875	0.872

Note: Bold values indicate the best performance across all evaluated methods for each metric.

Consistent with MCC performance, CONTRA-IL6 also achieved the highest F1-score of 0.549, representing an improvement of 4.3%–16.7% over existing methods (RAG_MCNIL6: 0.506; DGIL-6 and MVIL6: 0.498; UsIL-6: 0.487; StackIL6: 0.428; IL-6Pred: 0.382). This indicates better overall classification effectiveness, especially in addressing class imbalance, where optimizing both precision and SEN is critical. Additionally, CONTRA-IL6 also achieved a competitive SPE of 0.875, highlighting its robustness in correctly identifying non-IL-6-inducing peptides. Although its SEN of 0.767 was lower than that of some competing methods, it reflects a modest trade-off favoring a lower false-positive rate. In therapeutic screening contexts, minimizing false positives is often prioritized to reduce the risk of pursuing ineffective candidates. Despite this trade-off, CONTRA-IL6 maintained a high BACC (0.821) and a strong AUC (0.872), demonstrating reliable overall discrimination ability across all thresholds.

Moreover, we assessed statistical significance by performing paired bootstrap tests ($n=10\kern0.5em 000$ resamples) to compare the difference in the area under the precision–recall curve (AUPRC) between CONTRA-IL6 and the two reproducible methods (IL-6Pred and RAG_MCNNIL6). AUPRC was selected as the comparison metric because it is threshold independent and better suited for imbalanced datasets than AUC. As shown in Fig. S6, CONTRA-IL6 significantly outperformed both IL-6Pred (ΔAUPRC = 0.376, 95% confidence interval (CI): 0.290–0.468, $P<.001$) and RAG_MCNNIL6 (ΔAUPRC = 0.172, 95% CI: 0.085–0.266, $P<.001$). These results provide robust statistical evidence that CONTRA-IL6 achieves superior predictive performance over existing reproducible methods.

Collectively, these results demonstrate that CONTRA-IL6 provides the most balanced and accurate performance among the evaluated methods. Its marked improvements in both MCC and F1 highlight significant advances in predictive precision and generalizability.

### Model analyses and interpretation of CONTRA-IL6

#### Visualization of learned representations

To gain deeper insights into how different models learn class-discriminative features, we visualized the latent representations generated by the best single-feature model (ESM-1) and the proposed CONTRA-IL6 model (using the top-4 integrated features). We employed UMAP [26] to project high-dimensional feature vectors into 2D representations, facilitating intuitive interpretation. Additionally, KDE was used to visualize the local distribution densities, providing insights into the degree of separation between IL-6-inducing and non-inducing peptides.

**Figure 4:**
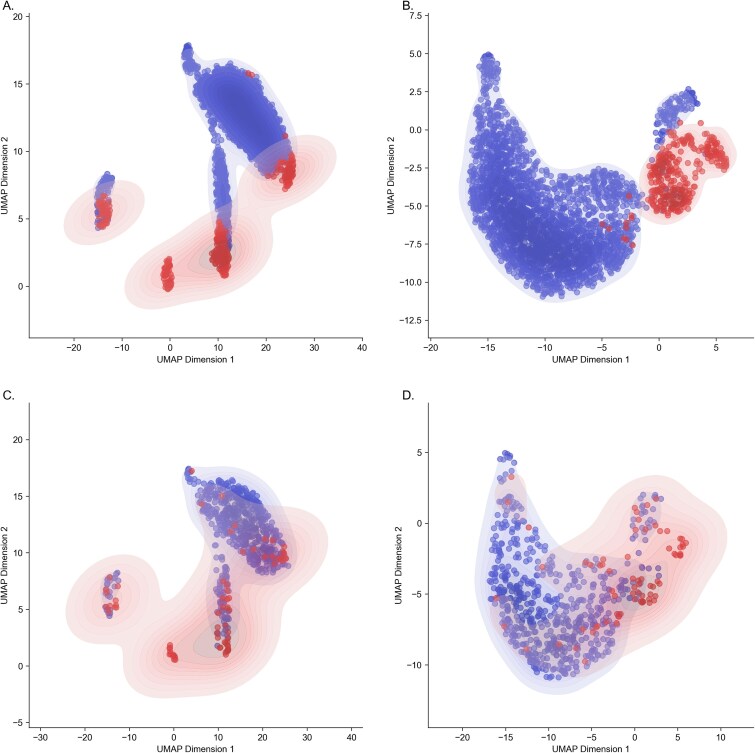
Uniform manifold approximation and projection and kernel density estimation visualizations of the training and independent datasets using the best single-feature model (ESM-1) and the CONTRA-IL6 model. (A) Visualization of the best single-feature model on the training dataset. (B) Visualization of the CONTRA-IL6 model on the training dataset. (C) Visualization of the best single-feature model on the independent dataset. (D) Visualization of the CONTRA-IL6 model on the independent dataset.

Figure 4 shows the UMAP and KDE visualizations, demonstrating distinct differences in representation learning between the two models. In the training dataset, the ESM-1 latent space appears relatively dispersed; positive samples are fragmented into three loosely connected clusters, while negative samples form two diffuse clusters (Fig. 4A). Although some separation between classes is evident, the broad and overlapping KDE contours suggest that ESM-1 has limited discriminative power in its learned embeddings. Conversely, CONTRA-IL6 shows a much more apparent separation on the same training dataset (Fig. 4B). Both positive and negative samples form more compact, well-separated clusters, indicated by tighter KDE contours. This reflects CONTRA-IL6’s stronger ability to learn class-discriminative features from multiple complementary sources.

On the independent dataset, the ESM-1 model’s feature representations become even more diffuse, with considerable overlap between IL-6-inducing and non-inducing samples (Fig. 4C). The KDE contours further indicate poor generalizability and a weaker ability to maintain class separation on unseen data. In contrast, CONTRA-IL6 maintained a relatively clearer separation between the two classes on the independent dataset (Fig. 4D). While some overlap is observed, as expected in a more challenging test scenario, the overall distribution and tighter KDE contours suggest improved generalization and robustness compared to ESM-1. These results highlight the benefits of using hybrid feature integration in CONTRA-IL6, which leads to more structured, interpretable, and generalizable representations that align with the model’s superior predictive performance. The visualization provides further evidence that CONTRA-IL6 not only fits well with training data but also retains its discriminative capacity on unseen examples.

#### Gradient-based model interpretation

**Figure 5:**
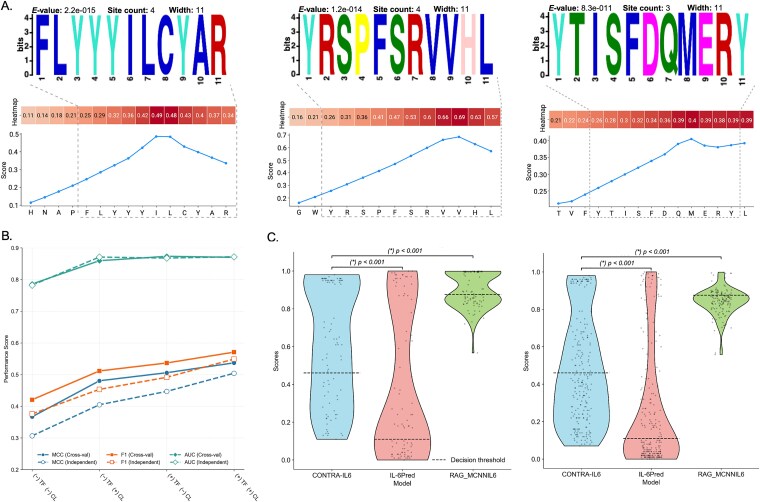
Comprehensive analysis of model interpretability and performance. (A) Visualization of gradient-based heatmaps and corresponding scores aligned with the top three motifs identified by the Multiple Expectation Maximizations for Motif Elicitation (MEME) tool, highlighting model focus regions associated with the motifs “FLYYYILCYAR,” “YRSPFSRVVHL,” and “YTISFDQMERY.” (B) Performance comparison of module ablation experiments on cross-validation and independent test datasets, where (+) indicates inclusion and (−) indicates exclusion of a module in the main architecture. (C) Distribution of prediction scores for CONTRA-IL6, IL-6Pred, and RAG_MCNNIL6 on positive and negative samples; statistical significance (*) is assessed using the Kolmogorov–Smirnov test between distributions.

To elucidate the underlying sequence features driving CONTRA-IL6’s predictions, we applied 1D-Grad-CAM++ [25], tailored for 1D representations. This approach enabled us to generate intuitive class activation heatmaps and gradient-based importance scores for pinpointing key amino acids influencing model predictions. Figure 5A shows three representative peptide sequences, each aligned with their top-ranked motifs identified using the multiple expectation maximizations for motif elicitation (MEME) [43] tool. Detailed parameters employed for MEME motif construction are summarized in Table S5. Our results strongly suggested that attention scores predominantly highlight the region “FLYYILCYAR,” which exactly matched the motif identified by MEME. Similar alignments were observed in sequence segments “YRSPFSRVVHL” and “YTISFDQMERY,” both of which exhibited strong agreement with MEME-derived motifs. This high concordance between attribution-based attention patterns and independently identified motifs suggests that the model captures recurring sequence features associated with IL-6-inducing peptides, rather than relying solely on arbitrary noise.

Furthermore, the gradient-based importance scores frequently showed stronger activations toward the C-terminal region of peptides, indicating that the model systematically assigns higher predictive weight to residues in this region. While attribution methods inherently reflect computational correlations rather than biological causality, this pattern suggests that C-terminal residues may harbor highly informative sequence signals that drive IL-6-associated immunogenicity.

To rigorously verify that this observation reflects meaningful sequence sensitivity rather than potential dataset artifacts, we conducted additional causality-oriented analyses using ISM and statistical validation, which systematically evaluate the effect of residue substitutions on model predictions. The detailed results of these analyses are provided in the Supplementary materials and Fig. S7.

Overall, these analyses provide interpretable insight into the computational decision patterns learned by CONTRA-IL6. By explicitly linking predictive weights to specific sequence features, this framework generates testable hypotheses about sequence motifs potentially associated with IL-6-linked immune responses, which can ultimately assist in the experimental validation of candidate immunomodulatory peptides.

#### Ablation study of modules

To quantitatively assess the individual contributions of key components within the CONTRA-IL6 architecture, we conducted a comprehensive ablation study focusing on the TF and CL modules. Model performance was evaluated across four distinct configurations: (i) Excluding the TF module: stacked embeddings were directly processed by the CL module. (ii) Excluding the CL module: only the CLS token from the TF output was used for classification. (iii) Excluding both modules: averaged stacked embeddings were directly fed into the final classifier. (iv) Integrating both modules: CONTRA-IL6 framework with both TF and CL modules.

Results show that the model without both modules achieved the lowest performance, as shown in Fig. 5B (detailed performance in Table S6), with an MCC of 0.367, F1 of 0.421, and AUC of 0.786 on the cross-validation set and MCC of 0.307, F1 of 0.376, and AUC of 0.782 on the independent dataset. The addition of either the TF or CL module resulted in significant performance improvements across all evaluation metrics. However, the model achieved the best result when both modules were integrated. This synergistic combination led to substantial gains: MCC scores improved by 3.1% to 17%, and F1s by 3.4% to 15%, during cross-validation. On the independent dataset, the corresponding improvements were even more pronounced, with MCC scores enhancing by 5.7% to 19.7% and F1s by 5.8% to 17.3%. These results demonstrate that integrating both TF and CL modules produces better results than using them separately, thus providing their synergistic role in improving model representation, robustness, and generalization.

To further evaluate the contributions of FL, residual connection, and CLS embedding, we independently removed or replaced them with standard alternatives. Specifically, FL was replaced with cross-entropy loss (CEL), residual connections were removed, and the CLS embedding was substituted with mean pooling over the entire sequence. As illustrated in Fig. S8, replacing FL with CEL resulted in the most significant performance degradation, yielding declines of 4.4% in MCC and 4.1% in F1 in cross-validation. This was followed by the removal of residual connections, resulting in a 3.8% decrease in both MCC and F1. Finally, using mean pooling instead of CLS embedding resulted in a 2.6% drop in MCC and a 2.5% drop in F1. These trends were consistent across the independent test set, with performance reductions ranging from 2.4% to 3.7% in MCC and 2.3% to 3.8% in F1. Collectively, these results validate the necessity of each component, confirming that their synergistic integration is critical and each module plays a non-redundant role in achieving the final state-of-the-art performance.

### Case study

#### Performance comparison on the newly external dataset

To further evaluate the generalizability and robustness of CONTRA-IL6, we constructed a newly external test dataset. This dataset included strictly defined negative samples that were experimentally validated as non-IL6 inducing by IEDB. We benchmarked CONTRA-IL6 against IL-6Pred and RAG_MCNNIL6, the only publicly available tools at the time of this study. Table 2 presents the performance comparison on this external dataset. CONTRA-IL6 consistently outperformed existing methods across all key metrics. Specifically, CONTRA-IL6 achieved an MCC of 0.334 and an F1 of 0.604. In comparison, IL-6Pred yielded significantly lower performance, with an MCC of 0.206 and an F1 of 0.538, while RAG_MCNNIL6 achieved an MCC of 0.222 and an F1 of 0.521. These results demonstrate that CONTRA-IL6 achieves stronger performance, surpassing the next-best method by 11.2% in MCC and 6.6% in F1.

**Table 2:** Performance comparison between CONTRA-IL6, IL-6Pred, and RAG_MCNNIL6 on the external test dataset.

Model	MCC	F1	ACC	BACC	SEN	SPE	AUC
IL-6Pred [16]	0.206	0.538	0.609	0.605	0.588	0.622	0.673
RAG_MCNNIL6 [22]	0.222	0.521	0.632	0.611	0.518	**0.704**	0.669
CONTRA-IL6 (Ours)	**0.334**	**0.604**	**0.677**	**0.670**	**0.635**	**0.704**	**0.710**

Note: Bold values indicate the best performance across all evaluated methods for each metric.

Analysis of prediction score distributions further highlighted differences in model behavior (Fig. 5C). CONTRA-IL6 showed a more balanced distribution, effectively distinguishing positive samples from negative ones. In contrast, IL-6Pred scores frequently clustered near 0.0 (indicating a false-negative bias in this dataset), whereas RAG_MCNNIL6 scores mostly clustered near 1.0 (indicating a false-positive bias). Moreover, the Kolmogorov–Smirnov test results confirmed that the distributional separation achieved by CONTRA-IL6 was statistically significant ($P<.001$). Collectively, these results indicate that CONTRA-IL6 offers a competitive advantage over existing methods on independent data. While the absolute performance metrics highlight the inherent difficulty of generalizing across diverse datasets, CONTRA-IL6 provides a more balanced classification profile than current tools, which suffer from severe trade-offs between sensitivity and specificity (e.g. high false-positive or false-negative rates). Thus, it represents a measurable step forward in predictive capability for this challenging task. To further demonstrate its practical utility, we also evaluated CONTRA-IL6 on SARS-CoV-2 proteins; these findings are discussed in the Supplementary materials and Tables S7–S17.

#### Prospective validation on novel food-derived peptides

To further assess the model’s applicability in a prospective setting, we evaluated its performance on a newly curated dataset of 19 food-derived peptides reported in recent literature (2024–26). This dataset challenges the model with sequences that are biologically distinct from the training set. Remarkably, CONTRA-IL6 correctly identified 9 out of 9 experimentally validated IL-6-inducing peptides. For instance, the model confidently predicted the activity of LPVGPLFN (score: 0.97) from *Tylorrhynchus heterochaetus* and LNEDELRDA (score: 0.97) from *Agaricus blazei*, both of which were experimentally shown to upregulate IL-6 in RAW264.7 cells. Conversely, when evaluating the non-IL-6-inducing peptides, the model correctly classified 4 out of 10 samples (scores <0.5). While our benchmark evaluation demonstrated the model’s overall high specificity, this prospective test reveals a highly sensitive profile when applied to the distinct sequence space of food-derived peptides. In the context of exploratory functional food research, this high-sensitivity profile is highly advantageous, as it minimizes the risk of discarding potentially novel bioactive hits (false negatives), while any false positives can be effectively filtered during subsequent experimental validation steps.

Collectively, these results establish CONTRA-IL6 as a highly capable predictor for initial screening of novel bioactive peptides. While the lower specificity on food-derived negatives suggests room for future improvement in differentiating subtle non-inducing motifs within this specific domain, the perfect recovery of positive hits underscores the model’s immense potential for accelerating the discovery of novel immunomodulatory peptides.

## Conclusion

In this study, we introduced CONTRA-IL6, a novel and interpretable hybrid DL framework specifically designed to accurately identify IL-6-inducing peptides. CONTRA-IL6 leverages a synergistic combination of TF module to capture global contextual representations and CL module for local sequence patterns extraction. Through a systematic and performance-driven model selection strategy, we identified multi-feature integration approach that significantly enhances the predictive performance. CONTRA-IL6 demonstrates state-of-the-art performance, outperforming existing approaches on independent evaluation. Notably, while UMAP and KDE visualizations illustrate distinct class separation in the learned feature space, the model’s interpretability is rigorously established through attribution and causal analyses. Moving beyond mere gradient-based attribution, this perturbation approach causally identifies functionally critical physicochemical constraints.

Despite its strength, CONTRA-IL6 has several important biological and computational limitations. Crucially, IL-6 induction is a highly complex, context-dependent process involving major histocompatibility complex presentation and specific cellular environments, rather than an isolated intrinsic property of a peptide. Current data inevitably collapses heterogeneous experimental conditions into a binary label. While stratifying data by assay type or species is biologically ideal, the severe scarcity of validated IL-6 peptides makes this computationally unfeasible, as further partitioning would cause extreme data fragmentation. Therefore, CONTRA-IL6 serves as a generalized first-line screening tool, estimating statistical immunogenic propensity rather than simulating context-independent biological responses. Furthermore, the current model is primarily trained on human- and mouse-derived data, limiting its generalizability across diverse host species. Therefore, broader and more diverse training datasets encompassing multiple species are necessary to improve the model’s predictive versatility. Moreover, CONTRA-IL6 specifically focuses on predicting IL-6-inducing peptides and lacks the ability to identify peptides that stimulate other cytokines, such as IL-2, IL-4, or IL-10. Furthermore, IL-6 induction can occur indirectly via complex immune pathways, such as through other interleukins or viral proteins (e.g. ORF8 in SARS-CoV-2), which may not be directly captured by peptide-level models. As part of our future work, we will expand the training dataset to cover a broader phylogenetic spectrum, enhancing cross-species applicability. We also plan to extend CONTRA-IL6 to support multi-cytokine prediction, enabling a more comprehensive view of peptide-mediated immune modulation. To capture indirect induction pathways and complex biological interactions, future work will incorporate protein-level modeling. Critically, we will integrate experimental validation loops to refine predictions and accelerate the translation of CONTRA-IL6 into practical applications for therapeutic peptide discovery.

**Key Points**

- We introduced a novel deep learning framework called CONTRA-IL6 for accurately predicting IL-6-inducing peptides.
- CONTRA-IL6 used a combination of Transformer fusion and convolutional localization modules to learn effective feature representations from stacked pretrained protein language model (PLM) embeddings.
- A robust predictive model has been developed, driven by attribution analyses, *in silico* mutagenesis, and feature space visualizations, significantly outperforming six state-of-the-art predictors when tested on an independent dataset.
- CONTRA-IL6 is now freely available as a standalone package at https://pypi.org/project/contra-il6/, providing an invaluable tool for large-scale screening in immunoinformatics research.

## Supplementary Material

Supplementary_Materials_Main_2_bbag250

Supplementary_Tables_bbag250

## Data Availability

The standalone package is available at https://pypi.org/project/contra-il6/. The open-source code, along with the benchmark and external test datasets, is freely accessible at https://github.com/cbbl-skku-org/CONTRA-IL6/. Any other data are available from the first and corresponding authors upon reasonable request.
