# Supplementary material for: CONTRA-IL6: an interpretable hybrid convolutional neural network and Transformer framework for accurate prediction of interleukin-6-inducing peptides using protein language models
Source: Brief Bioinform. 2026 Jun 11;27(3):bbag250. doi: 10.1093/bib/bbag250 (PMC13256231; doi:10.1093/bib/bbag250)
Supplement: Supplementary_Materials_Main_2_bbag250 [file supplementary_materials_main_2_bbag250.docx]

**Supplementary materials**

**Detailed description of 12 protein language models**

*Bepler*

The Bepler model is a deep learning framework that generates protein sequence embeddings encoding structural information. Using bidirectional LSTMs, it learns residue-specific vector representations through a dual feedback mechanism: global structural similarity between proteins and pairwise residue contact maps. A novel soft symmetric alignment (SSA) enables position-specific embeddings without direct positional correspondence. The model excels in predicting structural similarity and supports transfer learning for tasks like transmembrane domain prediction.

*Word2Vec*

Word2Vec is a technique for obtaining vector representations of words that capture semantic meanings based on surrounding words in large text corpora. It uses continuous Bag-of-Words (CBOW) or Skip-Gram models to map words to high-dimensional vectors, where semantically similar words are positioned closely.

*FastText*

FastText extends Word2Vec by incorporating subword information, representing words as bags of character n-grams. This allows FastText to handle out-of-vocabulary words effectively, making it particularly efficient for large-scale text processing.

*GloVe*

GloVe, or global vectors for word representation, focuses on local context. GloVe generates embeddings by factorizing a matrix of word-word co-occurrence counts from a large corpus, capturing both local and global context.

*SeqVec*

SeqVec uses the ELMo language model, to represent protein sequences as embeddings. Trained on the UniRef50 database, which contains a large set of protein sequences, SeqVec captures the biophysical properties of amino acid sequences, enabling predictions of secondary structure, disorder regions, and subcellular localization without evolutionary data.

*PLUS RNN*

PLUS RNN is a pre-training scheme called protein sequence representations learned using structural information (PLUS). It combines masked language modeling, a common NLP technique, with a protein-specific task called same-family prediction. This scheme is used to pre-train a bidirectional recurrent neural network (RNN), referred to as PLUS RNN, which learns representations incorporating structural information.

*ESM-1 (esm1_t34_670m_ur50s)*

ESM-1, with the variant esm1_t34_670m_ur50s, is a transformer-based model with 34 layers and 670 million parameters, trained on UniRef50. Using masked language modeling for self-supervised learning, it generates contextualized protein sequence embeddings for tasks like structure and function prediction.

*ESM-2 (esm2_t36_3b_ur50d)*

ESM-2, with the variant esm2_t36_3b_ur50d, features 36 layers and 3 billion parameters, trained on UniRef50. This advanced protein language model excels in predicting protein structure and function directly from individual sequences, supporting tasks like remote homology detection.

*ProtBERT (prottrans_albert_bfd)*

ProtBERT, part of the ProtTrans family, is a BERT-based model trained on the Big Fantastic Database (BFD). It uses self-supervised learning to generate contextualized protein sequence embeddings, capturing biophysical features for tasks like function prediction and structure analysis with high per-residue and per-protein accuracy.

*ProtALBERT (prottrans_albert_bfd)*

ProtALBERT, from the ProtTrans family, is based on the ALBERT architecture and trained on BFD. It generates embeddings capturing protein biophysical properties, suitable for tasks like secondary structure prediction and subcellular localization, without requiring labeled data.

*ProtXLNet (prottrans_xlnet_uniref100)*

ProtXLNet, part of ProtTrans, uses the XLNet architecture, an extension of Transformer-XL, trained on UniRef100. Designed for capturing long-range dependencies, it generates embeddings for protein sequence analysis, focusing on contextual relationships in large datasets via self-supervised learning.

*ProtT5 (protttrans_t5_xl_uniref50)*

ProtT5, based on the Text-to-Text Transfer Transformer (T5) architecture, is trained on UniRef50 using a BART-like masked language modeling denoising objective, masking 15% of amino acids. It captures essential protein features for tasks like function prediction and structure analysis.

**Optimization process**

*Loss function*

The benchmark dataset for IL-6-inducing peptide prediction is highly imbalanced across both training and independent test sets. Previous methods, such as StackIL6 [1] and UsIL-6 [2], addressed this issue using undersampling, in which the negative samples were divided into equal-sized subsets, each combined with the full set of positive samples to form balanced datasets. In contrast, MVIL6 [3] applied oversampling by duplicating positive samples to match the number of negatives.

While both approaches mitigate imbalance, they introduce notable limitations. Undersampling risks discarding valuable information by removing a portion of the majority class, potentially weakening the model’s generalization. On the other hand, oversampling can lead to overfitting, as the model may learn redundant patterns from repeated or synthetic samples rather than capturing meaningful distinctions.

In this study, we adopted Focal Loss (FL) [4] to address these limitations, a loss function specifically designed to handle class imbalance. FL down-weights easy, well-classified examples and emphasizes harder, misclassified ones, enabling the model to focus more effectively on difficult cases. The formulation of FL is as follows:

Here, $\alpha_{t}$ is a class-specific weighting factor, and $\gamma$ is the focusing parameter that controls the rate at which examples are down-weighted.

*Hyperparameter optimization*

To ensure optimal performance of the CONTRA-IL6 model, we conducted an extensive hyperparameter search. A 10-fold stratified cross-validation scheme was employed to preserve class distribution in each fold, thereby improving the robustness of the tuning process.

Model optimization was optimized using the AdamW [5] optimizer, which incorporates decoupled weight decay for improved generalization. Additionally, a learning rate scheduler was implemented to reduce the learning rate progressively across epochs, enhancing convergence stability. A detailed overview of the hyperparameter search space and selected values is provided in Table S4.

**Evaluation metrics**

In this study, we adopted the commonly used evaluation metrics: accuracy (ACC), balanced accuracy (BACC), sensitivity (SEN), specificity (SPE), Matthews correlation coefficient (MCC), and AUC. Additionally, we report the F1-score (F1) to offer a more nuanced assessment of model performance under class imbalance.

The metrics are defined as follows:

Where TP, TN, FP, and FN denote true positives, true negatives, false positives, and false negatives, respectively.

Furthermore, to statistically assess the difference between prediction score distributions, we performed the Kolmogorov–Smirnov (KS) test. This non-parametric test evaluates whether two samples are drawn from the same underlying distribution by measuring the maximum distance between their empirical cumulative distribution functions.

**Interpretation method**

To gain insights into the decision-making process of our model and identify which regions of the peptide sequences contribute most to the predictions, we employed 1D-Grad-CAM++ [6], an extension of the widely used Grad-CAM++ [7]. This adaptation enables visualization of class-discriminative regions in peptide sequences, thereby enhancing the interpretability of predictions made by our CL module.

The detailed procedure for computing the heatmap is presented below:

| **Algorithm 1. 1D-Grad-CAM++ for interpreting model.** |
| --- |
| **Input:** Input sequence $x$; Model output for target class $y^{c}$; Activation map from the target layer $A^{k}$; Epsilon $\epsilon= {10}^{-8}$ **Output:** 1D heatmap $h$  1. Compute the first-order gradient: $grad\leftarrow\frac{\partial y^{c}}{\partial A^{k}}$ 2. Compute the importance coefficient: $a_{c}^{k}\leftarrow\frac{\mathrm{grad}^{2}}{2\cdot\mathrm{grad}^{2}+\sum_{i} A^{k}\cdot\mathrm{grad}^{3}}$ 3. Compute the weight for each activation map: $\text{weigh}\text{t}\leftarrow\left\{ \begin{aligned} \mathrm{grad}\cdot a_{c}^{k}, \text{if grad}>0 \\ 0, \text{otherwise} \end{aligned} \right.$ 4. Interpolate the weights to match the input sequence length: $\text{weight.size}\leftarrow x.size$ 5. Generate the final heatmap by applying mix-max normalization: $h\leftarrow\frac{\text{weight}-min(\text{weight})}{\max\left( \text{weight} \right)-\min\left( \text{weight} \right)+\epsilon}$ |

This interpretability method provides a meaningful visual explanation by highlighting the subsequences that most strongly influence the model’s classification output, aiding in both model validation and biological interpretation.

**Hyperparameters analyses**

To assess the overall effectiveness of each value of a given hyperparameter in the CONTRA-IL6 framework, we fixed it at specific values and varied the remaining hyperparameters across multiple configurations. The results were then averaged to evaluate the marginal contribution of each fixed value in a generalized context. Fig. S4 summarizes the model’s performance in terms of MCC across multiple hyperparameter configurations over a 100-epoch training process. We evaluated the parameter $\gamma$ in FL [8] that influences model performance (Fig. S4A). Increasing $\gamma$ from 1 to 5 significantly improved MCC (from approximately 0.450 to nearly 0.470), suggesting that a stronger emphasis on hard-to-classify samples enables the model to learn more discriminative patterns. Next, we examined the effect of the target dimension $d_{\text{target}}$ in the FP module (Fig. S4B). Results showed only minor variation in MCC (ranging narrowly from 0.458 to 0.462), implying that dimensionality has a limited influence within the tested range. Notably, $d_{\text{target}}=256$ yields the best performance, suggesting that this dimensionality provides a good balance between expressiveness and regularization. Next, we investigated the number of encoder layers $L_{\mathrm{enc}}$ in the TF module (Fig. S4C). Increasing the number of layers has minimal impact on MCC, with performance peaking when $L_{\mathrm{enc}}=4$. This implies that deeper transformer architectures do not necessarily lead to better feature integration and may introduce complexity or potential overfitting.

Subsequently, we analyzed convolutional module-related hyperparameters. Fig. S4D shows the impact of varying $k$ within the CL module. A kernel size $k=5$ consistently achieved the best performance, likely because this size aligns well with the typical peptide length distribution (8–25 residues) and effectively captures local sequence patterns without excessive, fragmentation or smoothing. Next, we investigated the stride $s$ (Fig. S4E). Increasing the stride from 1 to 3 resulted in a slight but consistent MCC improvement, potentially by reducing redundant local information and providing a more efficient, coarse-grained representation of peptides. Finally, Fig. S4F examined the dilation $d$, and no significant difference was observed between the values of 1 and 3. This suggests that varying dilation in this module has a negligible effect, possibly because the default receptive field already captures sufficient context for peptide-level feature learning. Overall, our analyses underscore the importance of both FL weighting and carefully tuned convolutional configurations. Ultimately, these findings highlight the specific hyperparameter selections that yield optimal performance while simultaneously maintaining model simplicity and enhancing generalizability.

**IL-6 inducing peptides prediction in SARS-CoV-2 spike proteins**

The SARS-CoV-2 spike protein plays a central role in COVID-19’s development, notably contributing to the elevated levels of IL-6 observed in infected patients [9-12]. It is crucial to identify the IL-6-inducing peptides within this protein for understanding the cytokine storm and developing targeted therapeutic interventions.

In this study, we analyzed spike protein sequences from SARS-CoV-2 strains isolated in five different countries: India, China, the USA, Germany, and Italy, obtained from the prior study [13], originally sourced from the NCBI [14] database. From 1,259 peptides, our CONTRA-IL6 model identified 40 peptides as potential IL-6 inducers.

The top 20 predicted peptides were further evaluated by comparing CONTRA-IL6 predictions with two previously established IL-6 prediction tools, StackIL6 and IL-6Pred (Table S7). Notably, six peptides were consistently classified as IL-6 inducers by at least two models. Interestingly, three peptides, “NYNYLYRLFRKSNLK”, “LYRLFRKSNLKPFER”, and “NYLYRLFRKSNLKPF”, emerged as the most likely candidates to induce IL-6 production.

Our analysis beyond the spike protein to examine structural and non-structural proteins, including envelope protein, nucleocapsid phosphoprotein, membrane glycoprotein, ORF1ab, ORF3a, ORF6, ORF7a/7b, ORF8, and ORF10 (Tables S8–S17). Interestingly, our model did not identify any directly IL-6-inducing peptides within the ORF8 protein. This aligns with the prior research by Wu et al. [15], who reported that IL-6 induction via ORF8 occurs indirectly, by interacting with human IL-17 receptors, and subsequently activates downstream signaling pathways that lead to IL-6 expression. This highlights a key limitation of peptide-based DL models, which may not readily capture such an indirect mechanism. Although our computational predictions offer an informative starting point, experimental validation remains essential to confirm the IL-6-inducing potential of these peptides. Such validation would be invaluable for evaluating the inflammatory severity linked to specific viral strains or peptide candidates.

**Computational efficiency and hardware requirements**

All training and evaluation experiments for CONTRA-IL6 were conducted on a workstation equipped with an NVIDIA L40S GPU. The observed runtime on this hardware is summarized as follows (end-to-end training per fold):

- Training Speed: On the specified hardware, each training epoch requires approximately 5 seconds.
- Single-Fold Duration: The model reaches 100 epochs in approximately 8.3 minutes (500 seconds) per fold.
- Total Training Time: The complete 10-fold cross-validation process takes approximately 83.3 minutes (1.4 hours).

The use of L40S GPUs significantly accelerates the integration of multiple protein language models, allowing for efficient hyperparameter optimization and robust model evaluation.

**Training stability analysis**

To confirm that our model is not overfitting or exhibiting memorization artifacts, we visualized the training loss and validation MCC curves across 10-fold cross-validation. As shown in Fig. S5, CONTRA-IL6 demonstrates clear convergence, characterized by a steady decrease in training loss and a consistent improvement in validation MCC across 100 epochs. The validation performance exhibits minimal fluctuation, indicating stable optimization behavior and suggesting that the model does not suffer from training instability or overfitting.

**Causality, statistical validation, and biological relevance**

To address the concern that attribution methodologies do not inherently demonstrate causality or biological mechanisms, we performed a comprehensive *in silico* mutagenesis (ISM) to quantitatively assess the structural importance of the regions highlighted by our model. While UMAP/KDE and Grad-CAM++ serve as visualization and spatial attribution tools, ISM directly measures the effect of systematic amino acid substitutions on the model's output by calculating the change in predicted probability.

As shown in Fig. S7A, mutations introduced within the interior region toward the C-terminal end of true IL-6-inducing peptides caused a pronounced reduction in predictive probability, indicating that this region contributes substantially to the model’s prediction. In particular, substitution with proline (P) drastically reduced the predicted IL-6-inducing probability. This is consistent with the structural role of proline as an α-helix breaker that disrupts helical conformations by interfering with backbone hydrogen bonding and introducing structural kinks. In contrast, substitutions with isoleucine (I) and leucine (L) resulted in minimal changes or slight increases in scores, whereas most other amino acids caused substantial decreases. This trend was even more evident in the heatmap of IL-6-non-inducing peptides, where these residues markedly increased prediction scores. Such observations are biologically plausible because hydrophobic residues (I and L) help stabilize α-helical structures, while lysine (K), a positively charged residue, also shows favorable α-helix-forming propensity.

To rigorously prove whether the highlighted residues are causal drivers rather than random dataset artifacts, we employed the Non-IL-6-inducing dataset as a length-matched negative control. We extracted the mutational values exclusively from the critical region (positions 10–21) across both groups. A two-sided Mann-Whitney U test (Fig. S7B) revealed a highly significant downward shift in the mutational sensitivity of the IL-6-inducing group compared to the length-matched controls ($p < 0.001$), supporting that the C-terminal region identified by CONTRA-IL6 captures a non-random and prediction-relevant sequence pattern.

**Supplementary Tables**

Please refer to the accompanying “Supplementary Tables” Excel file for detailed data and results. The file contains the following tables:

**Table S1.** Detailed information on the external test dataset obtained from immune epitope database (IEDB).

**Table S2.** Detailed information of prospective validation dataset from food-derived peptides.

**Table S3.** Variants and dimensional details of the protein language models used.

**Table S4.** Ranges of the tuned hyperparameters.

**Table S5.** Parameters used for motif reconstruction in the multiple expectation maximizations for motif elicitation (MEME) tool.

**Table S6.** Detailed performance comparison of module ablation on cross-validation and independent dataset.

**Table S7.** Top 20 potential IL-6-inducing peptides predicted by our method compared with StackIL6 and IL-6Pred on spike proteins of SARS-CoV-2

**Table S8.** Prediction results for IL-6-inducing and non-IL-6-inducing peptides in the spike protein, comparing our method with StackIL6 and IL6PRED.

**Table S9.** Prediction of IL-6-inducing and non-IL-6-inducing peptides in envelope protein.

**Table S10.** Prediction of IL-6-inducing and non-IL-6-inducing peptides in membrane glycoprotein.

**Table S11.** Prediction of IL-6-inducing and non-IL-6-inducing peptides in nucleocapsid phosphoprotein.

**Table S12.** Prediction of IL-6-inducing and non-IL-6-inducing peptides in ORF1ab protein.

**Table S13.** Prediction of IL-6-inducing and non-IL-6-inducing peptides in ORF3a protein.

**Table S14.** Prediction of IL-6-inducing and non-IL-6-inducing peptides in ORF6 protein.

**Table S15.** Prediction of IL-6-inducing and non-IL-6-inducing peptides in ORF7a/b protein.

**Table S16.** Prediction of IL-6-inducing and non-IL-6-inducing peptides in ORF8 protein.

**Table S17.** Prediction of IL-6-inducing and non-IL-6-inducing peptides in ORF10 protein.

**Supplementary Figures**


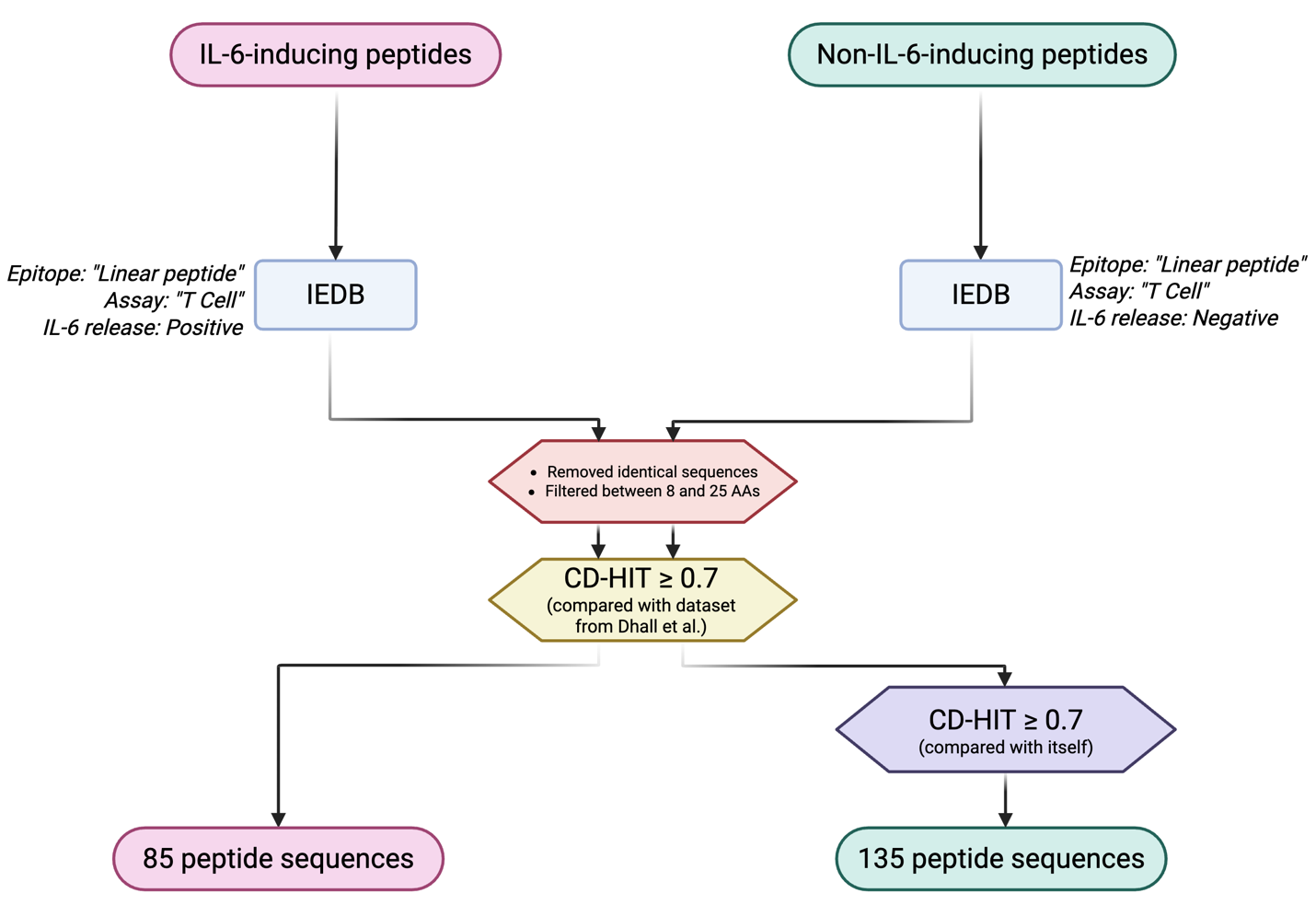


**Fig. S1.** Pipeline for collecting and preparing the external test dataset used in the case study.


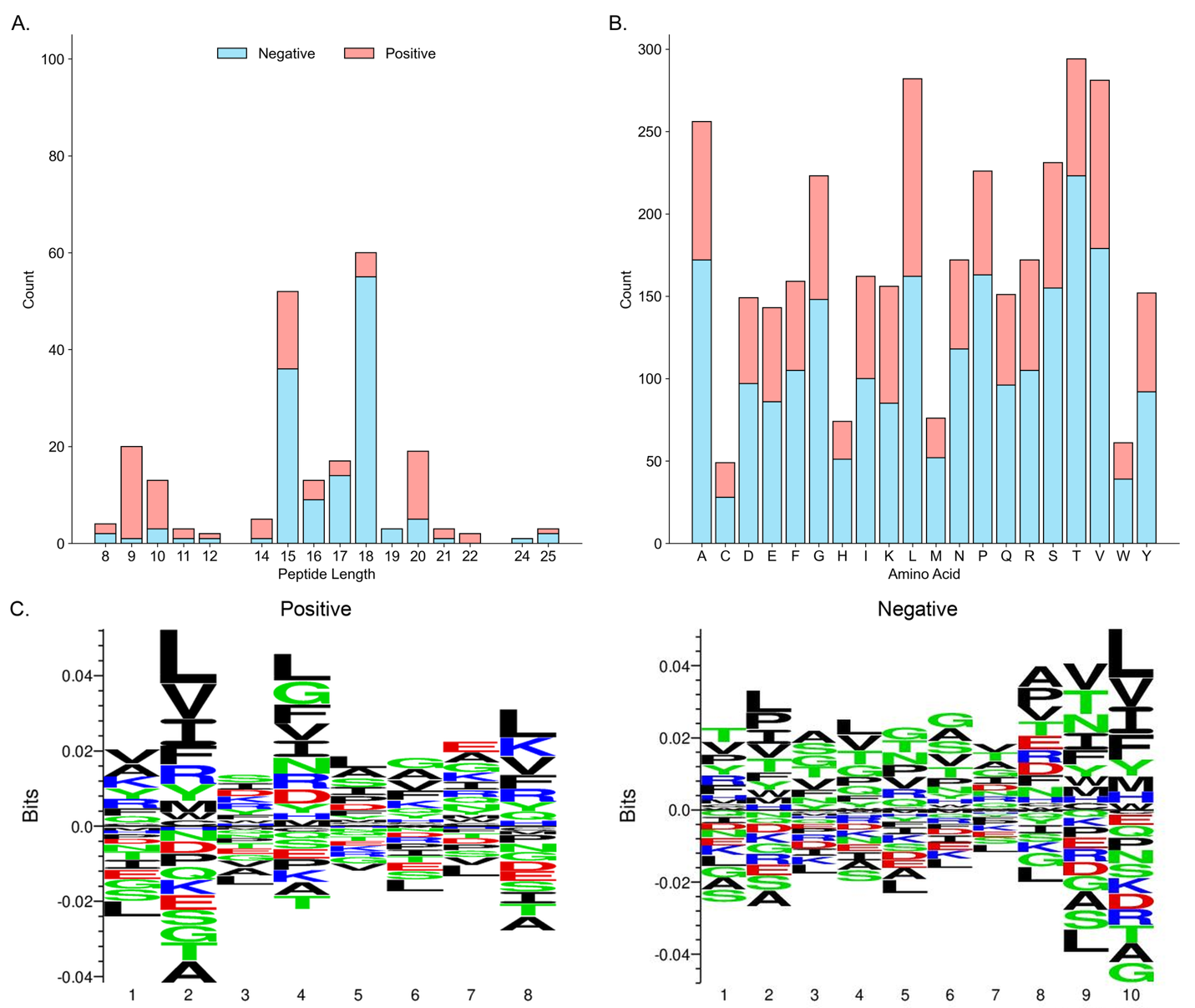


**Fig. S2.** External test dataset composition and sequence diversity analysis. (A) Distribution of peptide lengths in the dataset. (B) Overall amino acid frequency distribution across the dataset. (C) Sequence logos showing position-specific residue enrichment in positive (left) and negative (right) classes for the first 4 N-terminal and last 4 C-terminal residues.


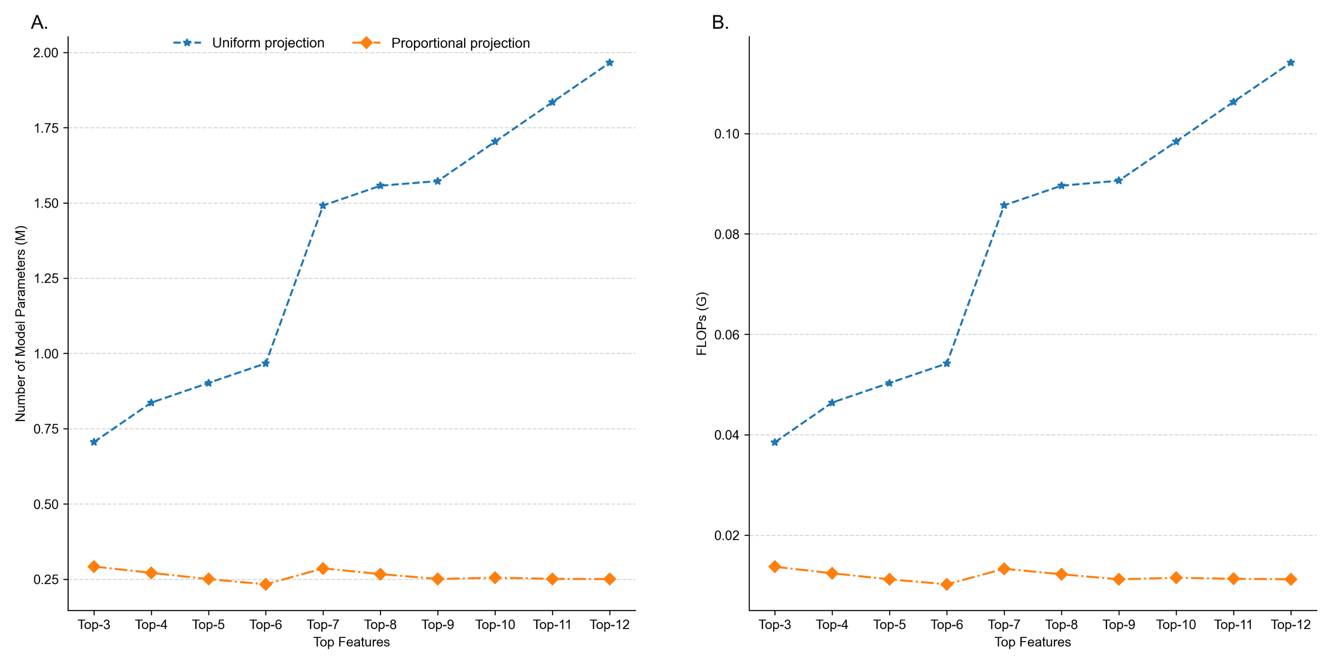


**Fig. S3.** Comparison of model complexity between two feature projection strategies, uniform projection and proportional projection, across various top-K experiments. (A) Number of trainable parameters (in millions). (B) Computational cost measured by floating-point operations (in gigaflops).

**
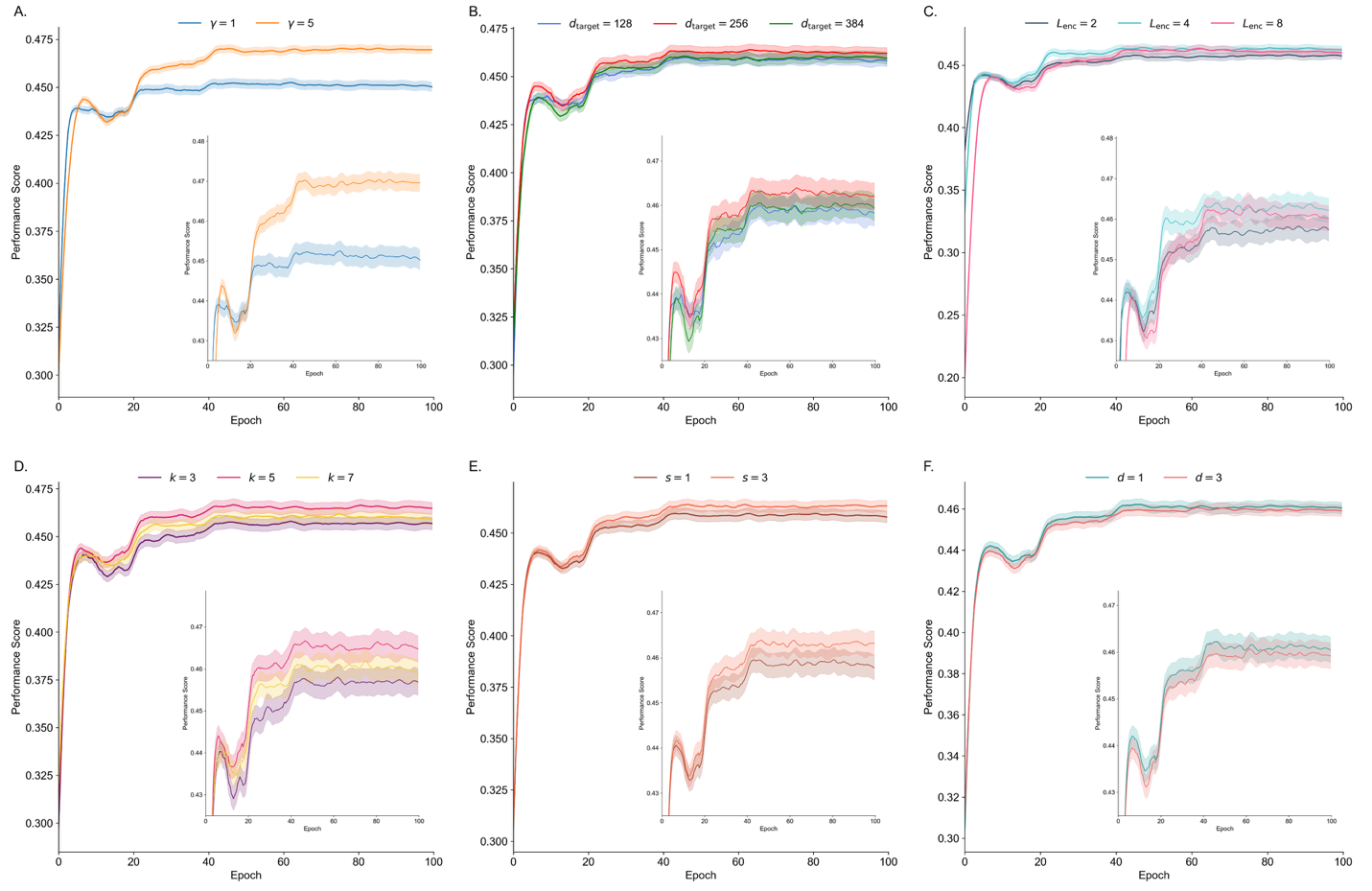
**

**Fig. S4.** Performance analysis of CONTRA-IL6 key hyperparameters based on the Matthews correlation coefficient over 100 training epochs. (A) Effect of the focusing parameter $\gamma$ in Focal Loss. (B) Effect of the target dimension $d_{\text{target}}$ in the Feature Projection module. (C) Effect of the number of encoder layers $L_{\text{enc}}$ in the Transformer Fusion module. (D) Effect of the kernel size $k$ in the Convolutional Localization (CL) module. (E) Effect of stride $s$ in the CL module. (F) Effect of dilation $d$ in the CL module. The insets highlight zoomed-in views for detailed analysis. The shaded error band represents the standard error across multiple configurations.


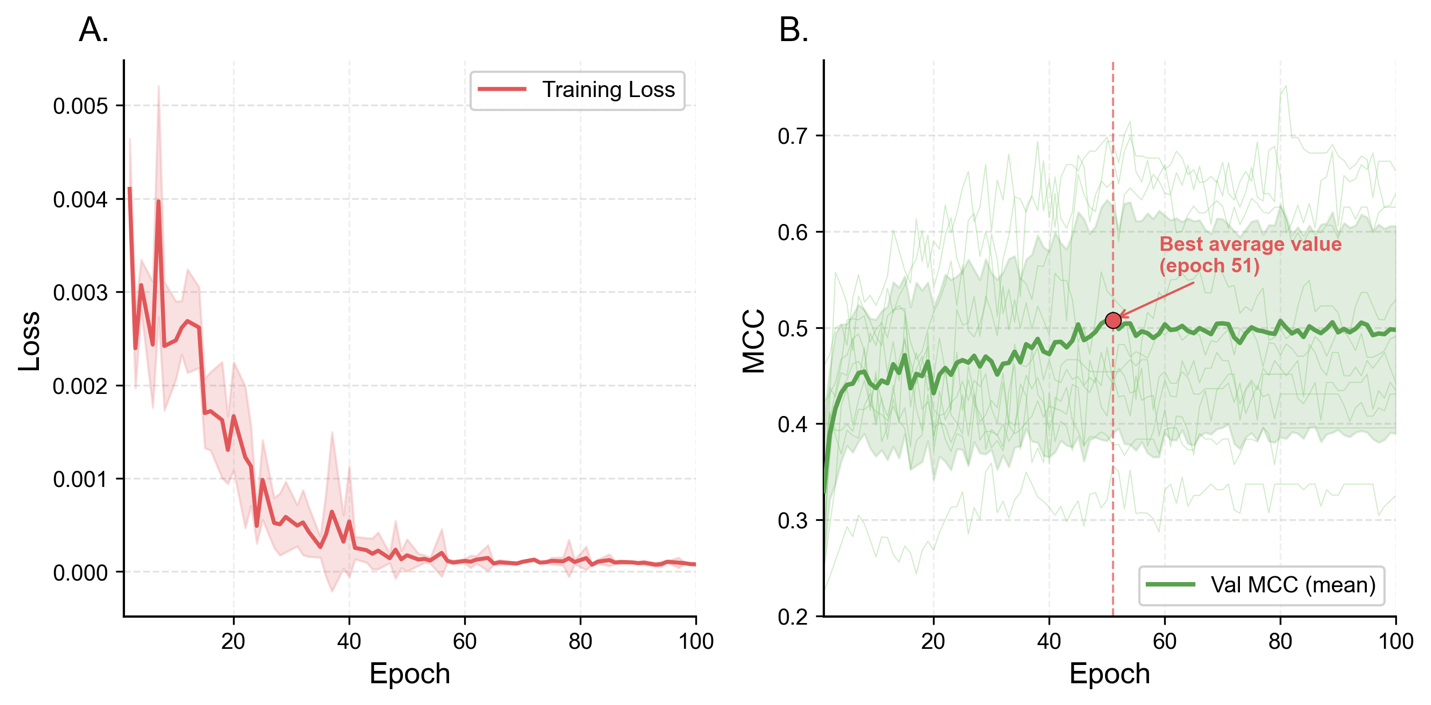


**Fig. S5.** Training stability analysis of CONTRA-IL6 across 10-fold cross-validation. (A) Training loss over 100 epochs, showing steady convergence. (B) Validation MCC across epochs, with the solid line representing the mean performance across folds and shaded regions indicating variability.


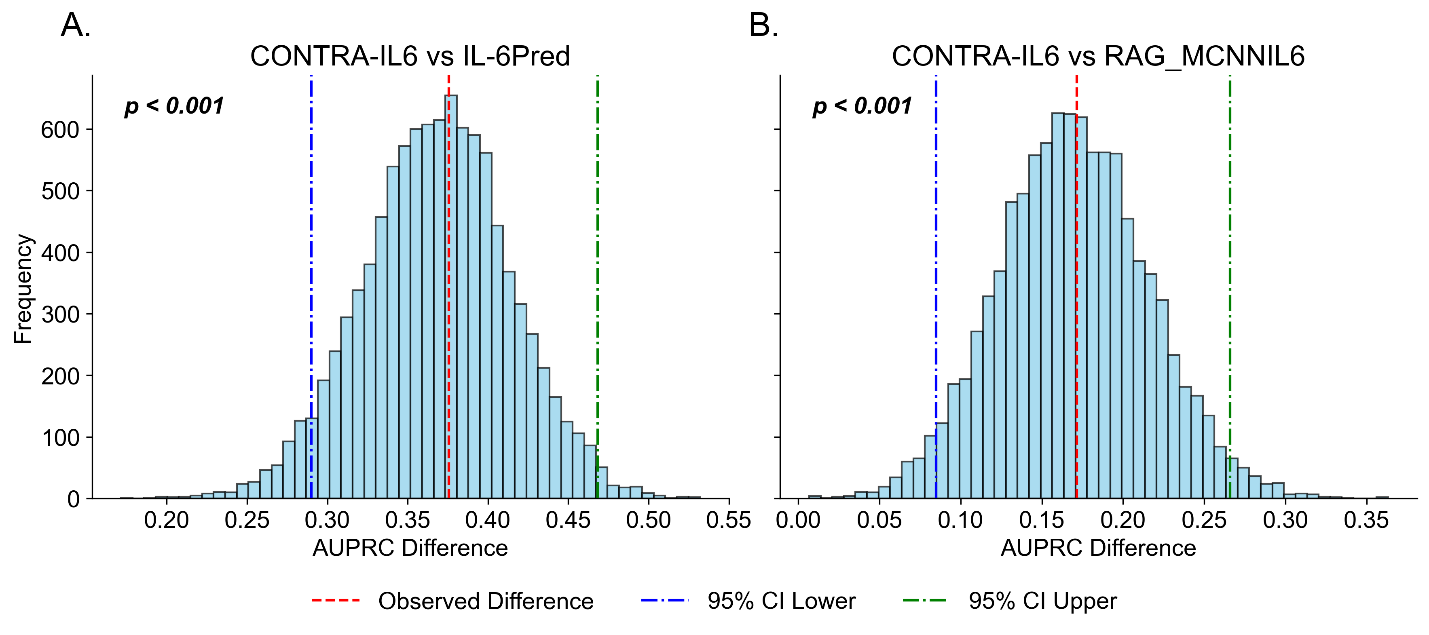


**Fig. S6.** Paired bootstrap test distributions of AUPRC differences between CONTRA-IL6 and baseline models. (A) Comparison with IL-6Pred. (B) Comparison with RAG_MCNNIL6. Histograms show the null distributions of AUPRC differences, with the observed difference indicated by the red dashed line and the 95% confidence interval bounds marked by blue (lower) and green (upper) dashed lines ($p < 0.001$ for both comparisons).


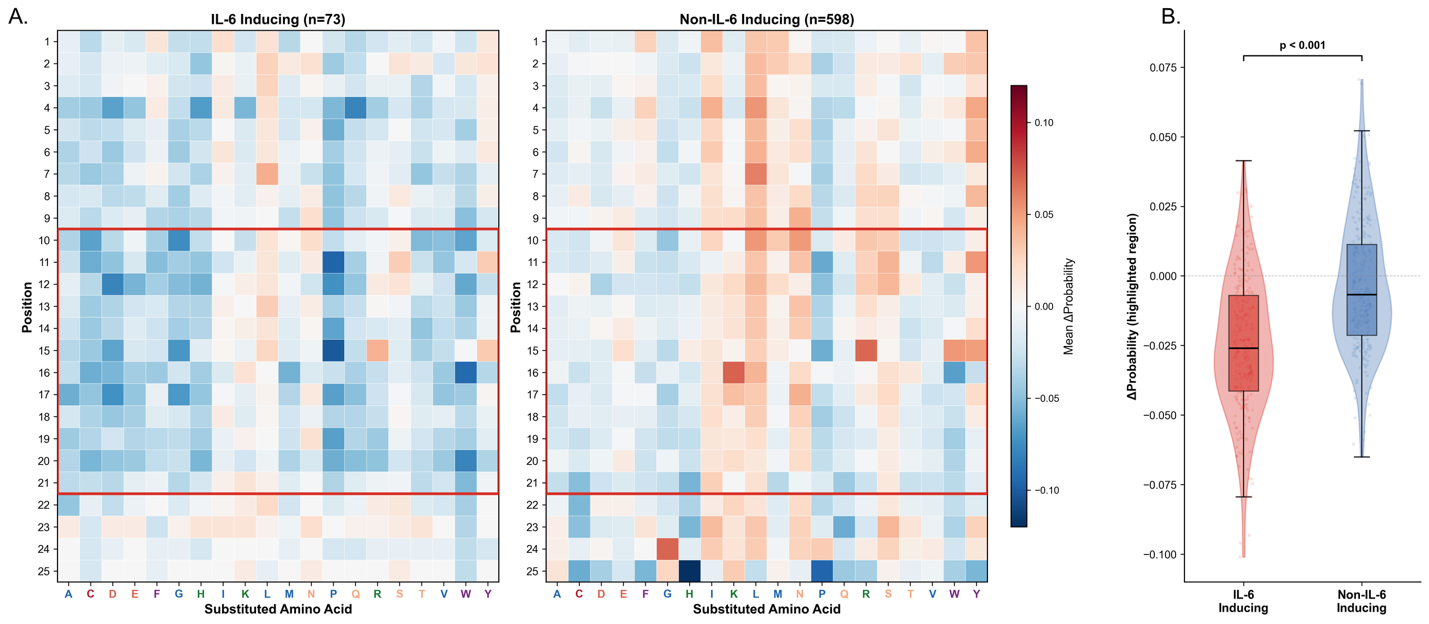


**Fig. S7.** Causality-oriented analysis using *in-silico* mutagenesis. (A) Aggregated mutational landscapes for IL-6-inducing and non-IL-6-inducing peptides. (B) Violin plots comparing the distributions of values extracted from the red-highlighted regions of both groups and statistical validation ($p < 0.001$).


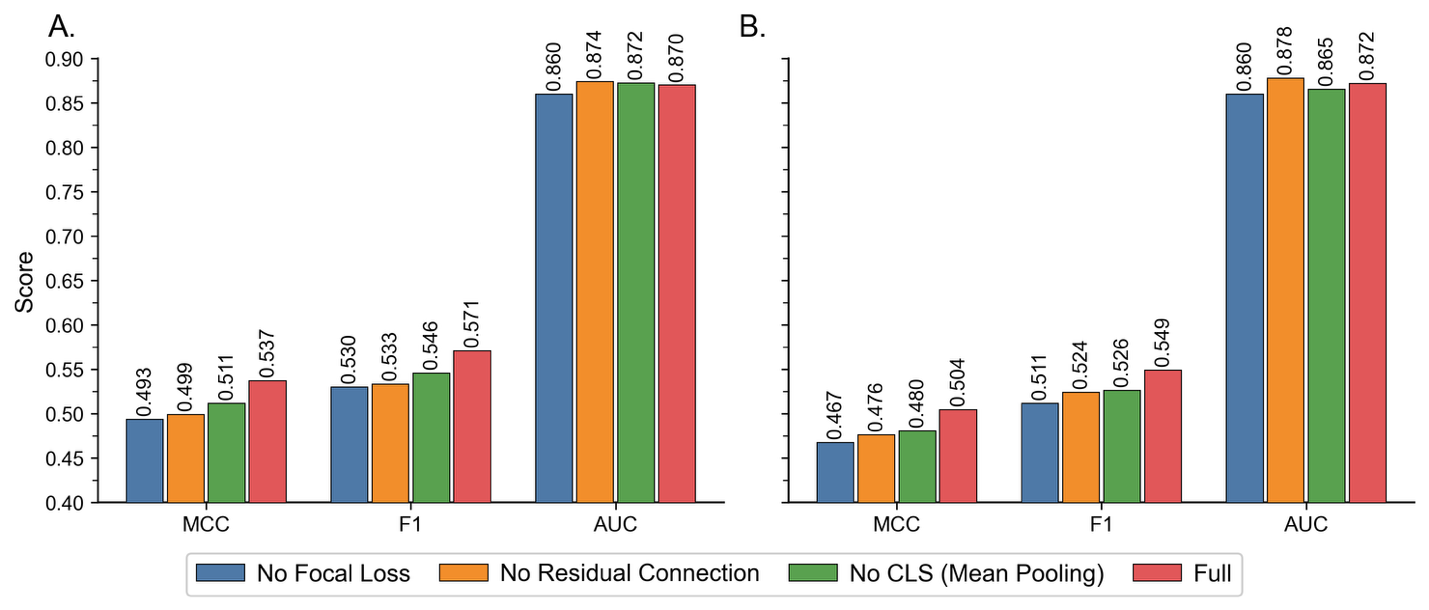


**Fig. S8.** Ablation study evaluating the contribution of key components to model performance. (A) Cross-validation results. (B) Independent test set results.

**References**

1. Charoenkwan, P., et al., *StackIL6: a stacking ensemble model for improving the prediction of IL-6 inducing peptides.* Briefings in Bioinformatics, 2021. **22**(6).

2. Liao, Y.-h., et al., *UsIL-6: An unbalanced learning strategy for identifying IL-6 inducing peptides by undersampling technique.* Computer Methods and Programs in Biomedicine, 2024. **250**: p. 108176.

3. Wang, R., et al., *MVIL6: Accurate identification of IL-6-induced peptides using multi-view feature learning.* International Journal of Biological Macromolecules, 2023. **246**: p. 125412.

4. Lin, T.-Y., et al. *Focal loss for dense object detection*. in *Proceedings of the IEEE international conference on computer vision*. 2017.

5. Loshchilov, I., *Decoupled weight decay regularization.* arXiv preprint arXiv:1711.05101, 2017.

6. Zhang, X., et al., *Fault diagnosis for small samples based on attention mechanism.* Measurement, 2022. **187**: p. 110242.

7. Chattopadhay, A., et al. *Grad-cam++: Generalized gradient-based visual explanations for deep convolutional networks*. in *2018 IEEE winter conference on applications of computer vision (WACV)*. 2018. IEEE.

8. Lin, T.Y., et al., *Focal Loss for Dense Object Detection.* Ieee Transactions on Pattern Analysis and Machine Intelligence, 2020. **42**(2): p. 318-327.

9. Wang, W., et al., *Up-regulation of IL-6 and TNF-α induced by SARS-coronavirus spike protein in murine macrophages via NF-κB pathway.* Virus Research, 2007. **128**(1-2): p. 1-8.

10. Patra, T., et al., *SARS-CoV-2 spike protein promotes IL-6 trans-signaling by activation of angiotensin II receptor signaling in epithelial cells.* Plos Pathogens, 2020. **16**(12).

11. Lee, A.R., et al., *SARS-CoV-2 spike protein promotes inflammatory cytokine activation and aggravates rheumatoid arthritis.* Cell Communication and Signaling, 2023. **21**(1).

12. Jugler, C., H.Y. Sun, and Q. Chen, *SARS-CoV-2 Spike Protein-Induced Interleukin 6 Signaling Is Blocked by a Plant-Produced Anti-Interleukin 6 Receptor Monoclonal Antibody.* Vaccines, 2021. **9**(11).

13. Dhall, A., et al., *Computer-aided prediction and design of IL-6 inducing peptides: IL-6 plays a crucial role in COVID-19.* Briefings in Bioinformatics, 2020. **22**(2): p. 936-945.

14. Sayers, E.W., et al., *Database resources of the National Center for Biotechnology Information.* Nucleic Acids Research, 2019. **47**(D1): p. D23-D28.

15. Wu, X., et al., *Viral Mimicry of Interleukin-17A by SARS-CoV-2 ORF8.* Mbio, 2022. **13**(2).
